# Factors Influencing Iron Levels Among Women of Reproductive Age and Children 6–48 Months in Livestock‐Keeping Communities in Narok County, Kenya

**DOI:** 10.1002/fsn3.4625

**Published:** 2025-01-22

**Authors:** H. K. Wakhungu, G. Abong, C. Muthike, N. Mutono, J. Muema, G. P. Omondi, S. M. Thumbi, Z. Bukania

**Affiliations:** ^1^ Department of Food Science, Nutrition and Technology University of Nairobi Nairobi Kenya; ^2^ Kenya Institute for Public Policy Research and Analysis Nairobi Kenya; ^3^ Feed the Future Innovation Lab for Animal Health Washington State University Pullman Washington USA; ^4^ Center for Epidemiological Modelling and Analysis University of Nairobi Nairobi Kenya; ^5^ Paul G. Allen School for Global Health Washington State University Pullman Washington USA; ^6^ Animal Health Innovation Lab, Department of Clinical Studies, Faculty of Veterinary Medicine University of Nairobi Nairobi Kenya; ^7^ Institute of Immunology and Infection Research, School of Biological Sciences University of Edinburgh Edinburgh UK; ^8^ Center for Public Health Research Kenya Medical Research Institute Nairobi Kenya

**Keywords:** child anaemia, haemoglobin, maternal anaemia, pastoralists

## Abstract

Proper nutrition is vital for maintaining good health for all people across their lifespan, especially children and mothers, who are especially vulnerable due to their specific nutrient needs. Despite the necessity of improved nutrition for these groups, some members do not fully meet their recommended daily micronutrient needs, a challenge exacerbated by different socioeconomic, cultural, and communal constraints resulting in malnutrition. Iron deficiency anaemia is a major concern among children and mothers, especially in pastoralist communities, due to poor nutrition and other related factors. Using a community‐based cross sectional study, this study investigated factors associated with hemoglobin levels among children and women in Narok County, Kenya. Anthropometrics were estimated using body mass index measurements for mothers, and the nutritional status of children was calculated using *Z*‐score measurements. Haemoglobin was measured using a rapid test (Hemocue 301). Multiple logistic regression models were fitted to assess the association between child and maternal risk factors and anaemia. Anaemia in children was associated with age (OR = 1.99, *p* = 0.047), pastoralism (OR = 2.25, *p* = 0.002), educational of the mother (OR = 0.74, *p* = 0.008), severe and moderate undernourishment (OR = 1.14, *p* = 0.049 and OR = 1.10, *p* = 0.023), respectively, not meeting children dietary diversity (OR = 1.18, *p* = 0.027), number of people in a household (OR = 1.84, *p* = 0.003), and maternal age (OR = 0.30, *p* = 0.010). On the other hand, the occurrence of anaemia in women was associated with pastoralism (OR = 2.22, *p* = 0.001), having a primary school level of education (OR = 0.51, *p* = 0.028), pregnancy status (OR = 5.36, *p* = 0.002), not meeting maternal dietary diversity (OR = 1.39, *p* = 0.026), number of household members (OR = 1.93, *p* = 0.023), age of the mother (OR = 0.53, *p* = 0.018), and having animals infected with East Coast Fever (*Theileria parva*) within the household (OR = 1.10, *p* = 0.023). The results highlight the multifaceted nature of malnutrition, specifically anaemia in pastoral households, with interventions aimed at reducing disease infections in cattle, improved household dietary diversity, and community health education geared towards maternal and child nutrition being best placed to improve the overall household health outcomes relating to anaemia.

## Graphical abstract

Anemia in children was influenced by children age, pastoralism, maternal educational level, children nutritional status especially severe and moderate undernourishment as well as not meeting children dietary diversity. Further, number of people in a household and maternal age significantly influenced anemia in children. For women of reproductive age, anemia was associated with practicing pastoralism, attaining primary school level of education, pregnancy status, not meeting dietary diversity, household size, their age, and having animals infected with East Coast Fever (*Theileria parva*).

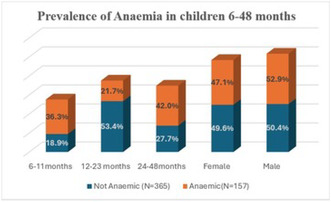

## 1 Introduction

Good nutrition is crucial in ensuring that individuals enjoy a healthy life underscoring the need to meet the recommended daily allowances (RDAs) in tandem with their physiological demands (World Health Organization 2021a, 2021b). Livestock ownership, although playing an important economic and nutritional role, especially in the provision of micronutrients, in livestock‐keeping communities (Iannotti and Lesorogol 2014; Hetherington et al. 2017; Otiang et al. 2022), can also be associated with an increase in all‐cause mortality (Kaur, Graham, and Eisenberg 2017).

Micronutrient deficiencies (MND) commonly known as hidden hunger, is one of the major challenges in developing countries (Branca et al. 2015). Paradoxically, although animal‐source foods are known to contain high levels of micronutrients (Siekmann et al. 2003; Ruel 2003; Grillenberger et al. 2006), livestock‐keeping communities have been shown to suffer from mixed levels of specific MND, including iron and folate (Wakhungu et al. 2024). This is majorly because a lot of people have access to the major staples, which are mostly carbohydrates leading to common MNDs such as vitamin A deficiency (VAD), zinc deficiency, and iron deficiency, especially in pregnant and lactating women as well as young children (Branca et al. 2015). Thus, despite access to some staple foods, inadequate nutrition breeds the triple burden of malnutrition (Muthayya et al. 2013; Branca et al. 2015).

**FIGURE 1:**
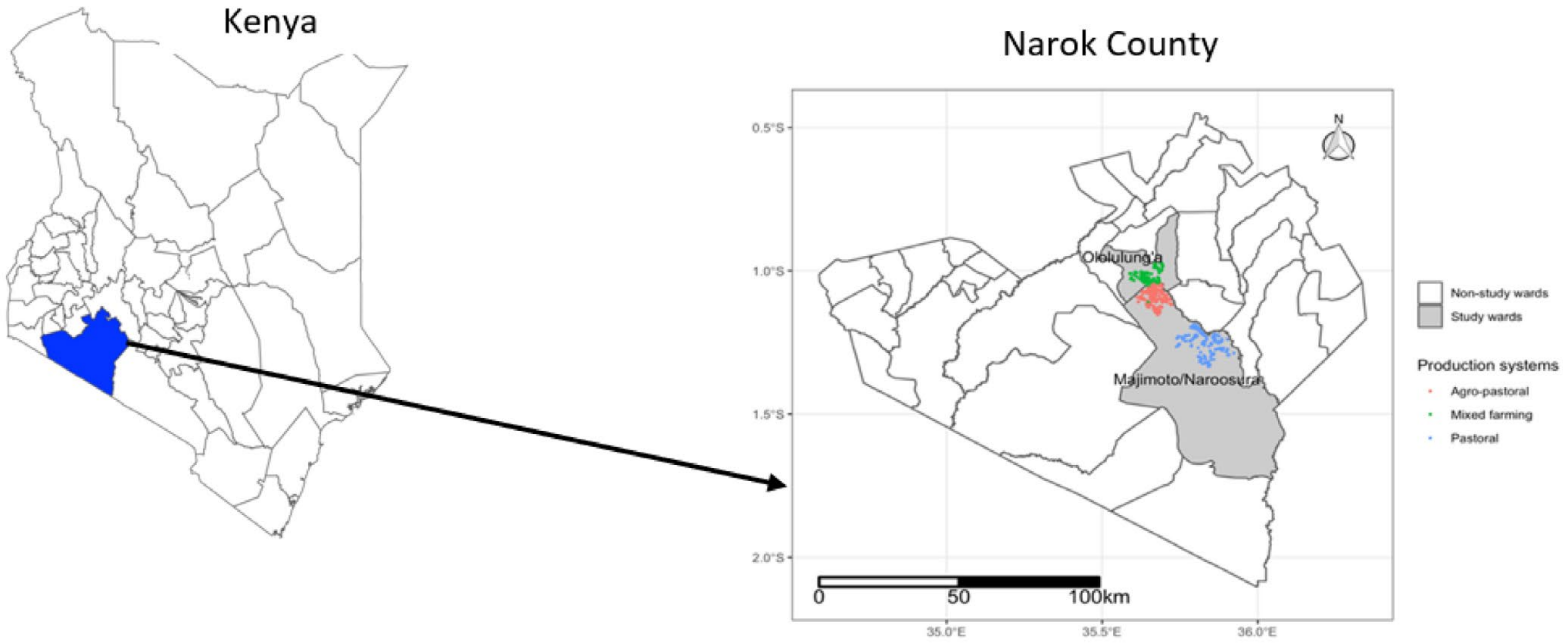
Map of Narok County showing the study sites (Shapefile source: GADM).

In Kenya, iron deficiency anemia is a major concern (Foote et al. 2013; Lemoine and Tounian 2020; Omuse et al. 2022) despite several initiatives aimed at the provision of iron supplements, especially among pregnant and lactating women and children below 5 years. Broadly, deficiency is exacerbated among pastoralist communities in part due to differential sociocultural practices, access to both formal and community health education, socioeconomic status, late initialization of antenatal care, nomadism, age—with young mothers being more at risk, low adherence to iron supplementation, and high levels of concomitant infections, especially gastrointestinal parasites (Iannotti and Lesorogol 2014; Lemoine and Tounian 2020; Njoroge, Mwangi, and Letourneau 2021; Wakhungu et al. 2024). However, there is still paucity in our understanding of how these factors, at the individual and community levels, interact to shape anemia outcomes among these populations to develop contextually viable preventive approaches to micronutrient deficiencies. Thus, the objective of this study was to explore correlates of anemia in pastoralist communities in Kenya and specifically elucidate on individual and community levels factors associated with anemia in livestock‐keeping communities in Narok West in southwestern Kenya (Figure 1).

## 2 Methodology

### 2.1 Study Site

This study was conducted in Oloolung'a and Naroosura/Maji Moto wards located within the larger Narok County, which is predominantly occupied by the Maasai community with the main economic activities varying from purely pastoral livestock keeping and tourism in the southeast and integrated crop agriculture and livestock keeping in the northwest following rainfall patterns (KNBS 2010, 2019; Bartzke et al. 2018). The two wards are located in the Narok South sub‐county which has an estimated 1.5 million cattle, sheep, and goats, and a human population that is largely rural (KNBS 2010, 2019). Narok County has reported high levels of iron deficiency anemia (Chepkorir, Bor, and Kipsaina 2019) with associated risk factors including age, parity, and education levels. Further, this region has reported high levels of early/teenage marriages and pregnancy, poor compliance with antenatal visits and nutritional interventions, low socioeconomic status, cultural and religious variations, and poor dietary patterns all risk factors for iron deficiency anemia (Chepkorir, Bor, and Kipsaina 2019; Lambrecht et al. 2021; Omuse et al. 2022; Wakhungu et al. 2024).

### 2.2 Data Collection and Analysis

Through a community‐based cross‐sectional study conducted between July and October 2022 in Oloolung'a and Naroosura/Maji Moto wards, 522 children and 525 mothers aged between 6–48 months, and 15–49 years, respectively, were randomly recruited into the study. Consenting households were included in the study based on the presence of a mother (lactating or non‐lactating), a child < 2 years of age, and household ownership of cattle.

For this study, data on demographic characteristics including level of education, occupation of the mother, child sex, nutritional status of the women—Body Mass Index (Kesari and Noel 2023), and minimum dietary diversity for mother and child (World Health Organization 2021a, 2021b) were collected. The nutritional status of children aged 6–48 months was calculated based on a weight‐for‐age anthropometric index (*Z*‐score) and categorized as severely undernourished (*Z*‐score < − 3.0), moderately undernourished (*Z*‐score −3.0 to −2.01) and Nourished (*Z*‐score ≥ −2.00) (World Health Organization 2005; Wang and Chen 2012). Data was also collected on household‐level predictors including East Cost Fever cases in the household, household number of people, Education level of household head, farming system, and household income.

Hemoglobin (HB) measurements were carried out using a rapid HB test (Hemocue 301), with children aged 6–48 months and women of reproductive age having HB concentration < 11 and < 12 g/dL considered anemic (World Health Organization 2021a, 2021b). In addition, a malaria test was also administered. Further, data on the Minimum Dietary Diversity for children (MDD‐C) and women (MDD‐W) were collected using a simple qualitative 24 h‐recall at the time of the household visit, respondents were asked about their dietary consumption based on 10 food groups [Grains, white roots, tubers, and plantains, Pulses (beans, peas, and lentils), Nuts and seeds, Dairy, Meat, poultry, and fish, Eggs, Dark green leafy vegetables, other vitamin A‐rich fruits and vegetables, other vegetables and other fruits], entered into the CommCare tool and later coded as per guidelines provided by the Food and Agricultural Organization of the United Nations (FAO and FHI 360, 2016). MDD‐W was then calculated as a summation of the different foods consumed by a household aggregated in the 10 predefined food groups, while MDD‐C was calculated based on their consumption of at least five of eight predefined food groups within the preceding 24 h of the visit. The index was calculated as a summation of the different foods consumed divided by the total number of children between 6 and 23 months whose data were collected. The consumption of animal‐source foods (ASF) in the household by children and women was scored if there was consumption of any food from an ASF source such as meat, milk, eggs, and fish (categories—Consumed ASF/Did not consume ASF). Summary statistics were calculated for the different variables based on the anemia levels in children and women of reproductive age (mother). Further, multiple logistic regression models were fitted to estimate the association between child and maternal risk factors and their contribution to anemia using R programming language (R Core 2013; Marschner, Donoghoe, and Donoghoe 2018). Before conducting multivariable analyses, univariate analyses were performed to identify predictors associated with children HB level and maternal HB (significance was assessed at *p* < 0.05). All variables with a *p*‐value less than 0.2 in the univariate regression analysis were included in the multivariable regression. Using the R programming (version 4.2.2) (R Core 2021), a logistic regression was carried out to identify predictors associated with HB level in women and children. This study was approved by the County Government of Narok, and the Kenya Medical Research Institute Scientific and Ethics Review Unit (Protocol No. SERU 4432), with written consent obtained from all participating households.

## 3 Results

### 3.1 Sociodemographic Characteristics

We sampled 525 mothers and 522 childrens from 525 unique households, with a mean household size of seven persons (95% CI [6.3–7.6]) and a mean household income of KES 16293.3 ± 780.5 (~126.30USD). Of these, 24.7% (129) reported cases of ECF in the last 1 year preceding the survey. Among the children, 48.9% (255) were male, with the overall mean age of the respondents' children being 23.3 (95% CI [22.3–24.4]) months. Approximately half of the children, 43.8% (229), were between 12 and 23 months of age. Over two‐thirds of the mothers interviewed were 35+ years (311), with 76.1% (397) lactating (Tables 1 and 2).

**TABLE 1:** Logistic regression model: risk factors for childhood anemia (Hb < 11 g/dL).

Dependent: anemic child		Not anemic (*N* = 365)	Anemic (*N* = 157)	OR (multivariable)
Child age (months)	6–11	69 (18.9%)	57 (36.3%)	
	12–23	195 (53.4%)	34 (21.7%)	1.99 (0.98–1.01, *p* = 0.047*)
	24–48	101 (27.7%)	66 (42.0%)	0.61 (0.32–1.32, *p* = 0.246)
Child sex	Female	181 (49.6%)	74 (47.1%)	
	Male	184 (50.4%)	83 (52.9%)	1.06 (0.69–1.64, *p* = 0.781)
Child Z‐score	Nourished	335 (97.3)	47 (29.9%)	
	Severely undernourished	2 (0.5%)	24 (15.3%)	1.14 (0.64–2.03, *p* = 0.049*)
	Moderately undernourished	8 (2.2%)	86 (54.8%)	1.10 (0.64–1.91, *p* = 0.023*)
Farming system	Agro‐pastoral	117 (32.1%)	37 (23.6%)	
	Mixed farming	153 (41.9%)	63 (40.1%)	1.67 (0.97–2.86, *p* = 0.064)
	Pastoral	95 (26%)	57 (36.3%)	2.53 (1.40–4.55, *p* = 0.002*)
Education (mother)	None	177 (48.5%)	77 (49%)	
	Primary	110 (30.1%)	47 (29.9%)	0.74 (0.42–1.31, *p* = 0.008*)
	Secondary	47 (12.9%)	23 (14.6%)	1.02 (0.50–2.09, *p* = 0.956)
	Tertiary	31 (8.5%)	10 (6.4%)	0.48 (0.18–1.23, *p* = 0.125)
Education (household head)	None	194 (53.2%)	78 (49.7%)	
	Primary	86 (23.6%)	44 (28%)	1.13 (0.66–1.95, *p* = 0.659)
	Secondary	61 (16.7%)	18 (11.5%)	0.65 (0.32–1.32, *p* = 0.236)
	Tertiary	24 (6.6%)	17 (10.8%)	0.72 (1.08–6.85, *p* = 0.034*)
Mother status	Lactating	278 (76.2%)	117 (74.5%)	
	Not pregnant and not lactating	67 (18.4%)	27 (17.2%)	0.93 (0.48–1.82, *p* = 0.841)
	Pregnant	20 (5.5%)	13 (8.3%)	0.93 (0.36–2.45, *p* = 0.029*)
Minimum dietary diversity for children	Met MDD	251 (68.8%)	113 (72%)	
	Not met MDD	114 (31.2%)	44 (28%)	1.18 (0.51–1.50, *p* = 0.027*)
Household number of people	Mean ± SD	7.4 ± 2.9	6.6 ± 2.4	1.84 (0.75–1.95, *p* = 0.003*)
East cost fever cases in the household	No	270 (74%)	123 (78.3%)	
	Yes	95 (26%)	34 (21.7%)	0.79 (0.47–1.32, *p* = 0.365)
Anemic mother	Anemic	56 (15.3%)	53 (33.8%)	
	Not anemic	309 (84.7%)	104 (66.2%)	0.34 (0.20–0.57, *p* = 0.001*)
Household income	Mean ± SD	16355.4 ± 593.5	17574.8 ± 947.6	1.00 (1.00–1.00, *p* = 0.040*)
ASF_Child	Consumed ASF	309 (84.7%)	132 (84.1%)	
	Did not consume ASF	56 (15.3%)	25 (15.9%)	0.61 (0.57–1.98, *p* = 0.021*)
ASF_Mother	Consumed ASF	111 (30.4%)	59 (37.6%)	
	Did not consume ASF	254 (69.6%)	98 (62.4%)	0.78 (0.46–1.33, *p* = 0.365)
Age mother bracket	19–24	24 (6.6%)	23 (14.6%)	
	25–28	55 (15.1%)	18 (11.5%)	0.30 (0.12–0.75, *p* = 0.010*)
	29–34	72 (19.7%)	20 (12.7%)	0.37 (0.16–0.89, *p* = 0.026*)
	35+	214 (58.6%)	96 (61.1%)	1.12 (0.46–2.72, *p* = 0.799)
BMI mother	Normal	195 (53.4%)	93 (59.2%)	
	Obese	32 (8.8%)	4 (2.5%)	0.29 (0.09–0.93, *p* = 0.037*)
	Overweight	84 (23%)	27 (17.2%)	0.71 (0.40–1.25, *p* = 0.231)
	Underweight	54 (14.8%)	33 (21%)	1.14 (0.64–2.03, *p* = 0.049*)

Abbreviations: BMI, Body Mass Index; Hb, hemoglobin; OR, odds ratio; SD, standard deviation.

* *p*‐value significant at *p* < 0.05.

**TABLE 2:** Logistic regression model: risk factors for maternal anemia (Hb) < 12 g/dL.

Dependent: anemic mother		Not anemic (*N* = 416)	Anemic (*N* = 109)	OR (multivariable)
Farming system	Agro‐pastoral	117 (28.1%)	38 (34.9%)	
	Mixed farming	163 (39.2%)	53 (48.6%)	1.05 (0.61–1.79, *p* = 0.867)
	Pastoral	136 (32.7%)	18 (16.5%)	2.22 (0.11–2.47, *p* = 0.001*)
Household head occupation	Employed full time	71 (17.1%)	24 (22.0%)	
	Businessperson	115 (27.6%)	22 (20.2%)	0.93 (0.48–1.82, *p* = 0.841
	Mixed farmer	199 (47.8%)	53 (48.6%)	0.78 (0.46–1.33, *p* = 0.365)
	Other	31 (7.5%)	10 (9.2%)	0.61 (0.27–1.40, *p* = 0.236)
Educational level of mother	None	211 (50.7%)	44 (40.4%)	
	Primary	115 (27.6%)	42 (38.5%)	0.51 (0.83–2.75, *p* = 0.028*)
	Secondary	59 (14.2%)	13 (11.9%)	0.85 (0.38–1.88, *p* = 0.684)
	Tertiary	31 (7.5%)	10 (9.2%)	0.32 (0.53–3.28, *p* = 0.555)
Educational level of household head	None	229 (55%)	44 (40.4%)	
	Primary	89 (21.4%)	43 (39.4%)	0.20 (1.69–5.34, *p* = 0.001*)
	Secondary	69 (16.6%)	10 (9.2%)	0.62 (0.27–1.40, *p* = 0.246)
	Tertiary	29 (7%)	12 (11%)	0.78 (1.07–7.22, *p* = 0.035*)
	Not pregnant and not lactating	73 (17.5%)	22 (20.2%)	
Mother status	Lactating	323 (77.6%)	74 (67.9%)	0.90 (0.48–1.70, *p* = 0.749)
	Pregnant	20 (4.8%)	13 (11.9%)	5.36 (1.89–15.19, *p* = 0.002*)
Minimum dietary diversity for women	Met MDD	120 (28.8%)	31 (28.4%)	
	Not met MDD	296 (71.2%)	78 (71.6%)	1.39 (0.77–2.50, *p* = 0.026*)
Number of people in the household	Mean ± SD	7.2 ± 2.7	6.8 ± 2.9	1.93 (0.83–1.05, *p* = 0.023*)
BMI of mother	Normal	228 (54.8%)	63 (57.8%)	
	Obese	31 (7.5%)	5 (4.6%)	0.63 (0.22–1.87, *p* = 0.409)
	Overweight	90 (21.6%)	21 (19.3%)	1.07 (0.56–2.02, *p* = 0.840)
	Underweight	67 (16.1%)	20 (18.3%)	1.67 (0.88–3.20, *p* = 0.019)
ECF case	No	316 (76%)	80 (73.4%)	
	Yes	100 (24%)	29 (26.6%)	1.10 (0.64–1.91, *p* = 0.023*)
Household income	Mean ± SD	16877.6 ± 780.5	15709.0 ± 782.1	1.00 (1.00–1.00, *p* = 0.912)
ASF consumption for mother	Consumed ASF	133 (32%)	38 (34.9%)	
	Did not consume ASF	283 (68%)	71 (65.1%)	1.00 (0.57–1.74, *p* = 0.999)
Age of the mother	19–24	35 (8.4%)	14 (12.8%)	
	25–28	59 (14.2%)	14 (12.8%)	0.41 (0.15–1.13, *p* = 0.086)
	29–34	75 (18%)	17 (15.6%)	0.53 (0.21–1.37, *p* = 0.018*)
	35+	247 (59.4%)	64 (58.7%)	1.09 (0.41–2.91, *p* = 0.868)

Abbreviations: ASF, animal sourced foods; BMI, Body Mass Index; Hb, hemoglobin; OR, odds ratio; SD, standard deviation.

* *p*‐value significant at *p* < 0.05.

More than half 58.8% (307) of the mothers did not meet the minimum dietary diversity and even more children 364 (69.7%) not meeting the minimum dietary diversity. Majority of the children 84.5% consumed at least one ASF (441). In contrary, 352 (67.4%) of the mothers did not consume at least one ASF 1 week prior to the survey date. On nutritional status, more than half of the children 382 (73.2%) > 3 Z‐score with a few 25 (4.9%) severely undernourished (−3 Z‐score). For mothers, 16.7% (87) were underweight (BMI < 18 Kg/M^2^).

One‐half of the mothers in this study, 48.8% (255) had no formal education, while 30.1%, 13.7%, and 7.9% had primary, secondary, and tertiary education, respectively (Table 2). Over half of the households' heads had no formal education 65.7% (343), with 7.9% (41) of them having attended college/university.

### 3.2 Association Between Children, Maternal and Household Factors and HB Level of Children and Mothers

From the univariable analysis, child age (months), child sex, nutritional status (child z‐score), farming system, education level of mother, education level of household head, mother status, MDD‐C, MDD‐W, household number of people, anemia status of the mother, household income, ASF_Child, ASF_Mother, age of the mother, BMI status of the mother variables had a *p*‐value less than 0.2 and were included in the multivariate analysis. In the multivariable models, the significant risk factors associated with anemia in children were child age, child sex, nutritional status, farming system, maternal education, education level of the household head, maternal anemia status, household size, household income and consumption of animal source foods (ASF‐Child). For maternal HB level, maternal anemia status, farming system, maternal education, ECF status, household status and maternal nutritional status were associated with an increased risk of maternal anemia.

### 3.3 Risk Factors for Children and Maternal Anemia

The results of this study indicate that child age was a significant factor in the likelihood of developing anemia, with a higher prevalence of anemia among children between the ages of 12–23 months compared to those between 24 and 48 months showing higher odds (OR = 1.99, *p* = 0.047) (Table 1). The sex of the child was not found to be a significant factor (OR = 1.06, *p* = 0.781). The nutritional status of children had a significant association with anemia, where moderately undernourished children had increased odds of anemia (OR = 1.10, *p* = 0.023), and severely undernourished children were at an even greater risk (OR = 1.14, *p* = 0.049).

Children in pastoral farming systems had significantly higher odds of anemia compared to those in agro‐pastoral systems (OR = 2.53, *p* = 0.002). Maternal education was found to be protective, with children from mothers who had completed primary education having lower odds of anemia (OR = 0.74, *p* = 0.008). Additionally, the household head's tertiary education was associated with reduced odds of anemia in children (OR = 0.72, *p* = 0.034). Larger household size was positively correlated with the risk of childhood anemia (OR = 1.84, *p* = 0.003), while children of anemic mothers had higher odds of being anemic (OR = 0.34, *p* < 0.001). Household income had a slight effect on anemia risk (OR = 1.00, *p* = 0.040), and the consumption of animal‐sourced foods (ASF) was significantly associated with reduced anemia level (OR = 0.61, *p* = 0.021).

For maternal anemia (Table 2), pastoral farming increased the risk (OR = 2.22, *p* < 0.001), while maternal education was associated with reduced odds (OR = 0.51, *p* = 0.028). The study findings also showed that maternal status such as being pregnant had significantly higher odds of anemia compared to the lactating mothers (OR = 5.36, *p* = 0.002). When the household size and nutritional status of the mother were assessed, it was found that larger household size (OR = 1.93, *p* = 0.023) and underweight status (OR = 1.67, *p* = 0.019) were also associated with an increased risk of maternal anemia. The presence of ECF in the hard (OR = 1.10, *p* = 0.023) was also associated with increased risk of maternal anemia.

## 4 Discussion

The results of this study highlight several important factors influencing anemia among children 6–48 months and mothers/women of reproductive age (15–49 years). The finding that child age is a significant factor in the likelihood of anemia supports existing research, including that of Msaki et al. (2022), which suggests that younger children, particularly those under 2 years of age (< 24 months), are more susceptible to anemia due to their growth needs and an increased fetal erythropoietic activity resulting in a low iron storage state (Choi, Kim, and Pai 2000; Wieringa et al. 2007), lower iron absorption, larger intestinal iron loss, and more frequent infections. Therefore, dietary sources of iron are vital in keeping up with the rapid rate of erythropoiesis, which may result in anemia if the dietary iron sources are inadequate. However, the lack of a significant association between child sex and anemia contrasts with the findings of Melku et al. (2018), who noted that male children have a higher risk of anemia due to higher metabolic demands and testosterone‐driven erythropoiesis. Male children have higher levels of testosterone than female children during their pre‐puberty stage. Testosterone hormone is a stimulator of the production of red blood cells (erythropoiesis) and increased body metabolism. Therefore, high levels of this hormone among boys cause these children to have a higher nutrient requirement than girls, thus inadequate nutrient intake places boys at a higher risk of anemia than girls.

Undernutrition was strongly associated with anemia, particularly in children who were moderately or severely undernourished. These findings are consistent with studies conducted in Ethiopia (Ahmed, Hossain, and Sanin 2013; Gebremeskel et al. 2020), which have shown that undernutrition, especially stunting and wasting, is a major risk factor for anemia. These results underscore the common risk factors in developing regions, where poor dietary intake, limited healthcare, and socioeconomic factors contribute to both undernutrition and anemia in children.

The higher odds of anemia in children from pastoral communities (OR = 2.53) compared to agro‐pastoralists reflect the challenges faced by pastoralist populations in accessing diverse diets and healthcare services. This is in line with the work of Geletaw et al. (2021), who reported similar findings in Ethiopian pastoralist communities. The harsh and unfavorable environmental conditions which are mainly based solely on livestock herding are a significant contributor to poor health outcomes and consequently anemia of children in pastoralist communities. On the other hand, children from agro‐pastoral systems tend to have better health and nutrition outcomes due to access to diverse diets as a result of crop cultivation and reliance on non‐pastoral income‐generating activities by their parents (Megersa, Haile, and Kitron 2021).

Maternal education played a protective role against child anemia, a result that mirrors the findings of studies in Jordan and other developing countries (Al‐Suhiemat, Shudifat, and Obeidat 2020; Chandran and Kirby 2021) which found that children from mothers with low education levels were more likely to have anemia compared to their counterparts with higher levels of education. The lower odds of anemia in children whose mothers had primary education suggest that interventions aimed at increasing maternal education could significantly improve child health outcomes. Elsewhere, higher maternal education has been associated with better nutrition knowledge, diverse diets and ultimately better health outcomes (Chandran and Kirby 2021; Mutuku, Ochola, and Osero 2020). In developing countries, especially among pastoralist communities, cultural practices characterized by food restrictions and taboos are highly followed which end up altering children's dietary intake (Motadi et al. 2020; Shrestha et al. 2021; Walters, Bendulo, and Stoecker 2019). Similarly, the use of herbs and traditional medicine with very minimal dependence on proper healthcare services contribute to a lack of adherence to immunization schedules among children, limited access to deworming and consequently increased morbidity and anemia among these children.

Similarly, the reduced odds of anemia in children from households with educated heads reflect the critical role of socioeconomic factors in health outcomes, a conclusion supported by Vallières et al. (2013). It has been seen that household heads usually provide the financial means for meeting the households' needs, especially in pastoralist communities where the women are mainly housewives and are expected by their culture to stay at home and care for the children. Therefore, the household head's education level is a key determinant in the decisions that he/she will make concerning the spouse and children's health care, diet and well‐being that influence their nutritional and health status.

The finding that consuming ASF slightly reduce the risk of anemia highlights the need for further investigation into the quality and safety of these foods, particularly in pastoralist communities, where access to safe and nutritious foods may be limited. This is probably due to the effect of animal‐based pathogens present in ASFs especially if the foods are not well stored, properly prepared or even assessed and tested by food safety officials during slaughter. Therefore, if children consume such ASFs it might exacerbate anemia by exposing the children to illness and inflammation caused by the pathogens (Lambrecht et al. 2021). Another probable reason for this could be, the presence of intestinal parasites among the children which lead to helminthic infections, diarrhea, dysentery and intestinal malabsorption (Palacios et al. 2021). Thus, despite the consumption of ASF, if the children have these parasites, they will still be more susceptible to anemia.

The weak association between household income and anemia risk, while unexpected, may indicate that other factors, such as household size and maternal health, play more direct roles in influencing anemia. This finding is consistent with the metanalysis of studies carried out in Southern Asia and Sub‐Saharan countries (Gebrie and Alebel 2020; Sunuwar et al. 2023; Tesema et al. 2021). This could probably be because a low household income is strongly correlated with food insecurity which consequently results in limited access to iron‐rich foods and other micronutrient‐dense foods for children thus increasing their risk of developing anemia (Chandran and Kirby 2021; Geletaw et al. 2021; Nambiema, Robert, and Yaya 2019; Tesema et al. 2021). Most of the households with low income tend to live in areas with poor living conditions limiting their access to proper water and sanitation which in turn leads to increased infections among children and consequently increased risk of anemia (Bharati 2015). Furthermore, households with low income are less likely to afford quality healthcare services for their children during illnesses (Tesema et al. 2021).

For maternal anemia, the significant association with pastoral farming practices aligns with Roba et al. (2015), who also found that women in pastoral communities are at higher risk of anemia due to limited dietary diversity and healthcare access. The heightened risk of anemia among pregnant women reflects the increased nutritional demands during pregnancy and the common issue of poor adherence to iron supplementation in these communities (Gebremariam et al. 2019). Pregnant women are the most vulnerable to anemia in developing countries especially due to the high poverty rates, high food insecurity status and poor adherence to iron and folic acid supplementation among these women (Gebremariam et al. 2019; Kamau, Mirie, and Kimani 2018; Lyoba et al. 2020; Saragih et al. 2022; Sendeku, Azeze, and Fenta 2020). In pastoral communities like the case of this study, food taboos and restrictions for pregnant women due to cultural beliefs are among the leading causes of malnutrition and increased risk of anemia in pregnancy (Chakona and Shackleton 2019; Walters, Bendulo, and Stoecker 2019). These taboos also result to not meeting the minimum dietary diversity which was also found to significantly increase the odds of anemia in this study. Furthermore, other studies have shown a high prevalence of undernutrition among pregnant women in pastoral communities due to the prolonged droughts leading to limited access to food, low dietary diversity and consequently micronutrient deficiencies (Birara Aychiluhm, Gualu, and Wuneh 2022). On the other hand, lactating mothers were less likely to have anemia probably due to lactational amenorrhea whereby iron loss as a result of breastfeeding is relatively lower than that occurring during menstruation in non‐pregnant and non‐lactating women (Girma et al. 2020).

Larger household sizes and maternal underweight status also emerged as significant factors associated with maternal anemia. This findings are consistent with studies in Bangladesh and Ethiopia (Kamruzzaman 2021; Kedir, Nebi, and Bereka 2023), which have highlighted the compounded effects of poverty, high fertility rates, and malnutrition on maternal health. This is especially so if the mother has many children, a higher parity with very short birth intervals is a risk factor for anemia among mothers (Owais et al. 2021). It was also noted that underweight mothers had significantly higher odds of anemia than those with a normal nutrition status. This is consistent with the findings of the studies carried out in Bangladesh and Ethiopia (Kamruzzaman 2021; Kedir, Nebi, and Bereka 2023). Undernutrition is mainly caused by undernourishment; this inadequate dietary intake might explain the higher chances of anemia among underweight women. Underweight women likely have inadequate access to dietary iron intake (Kamruzzaman 2021). Infestation of East Coast fever (ECF) in the community significantly increased the odds of anemia among the women. This could be probably due to the fact that ECF results in high mortality of animals within the pastoralists households which reduces their capacity to slaughter their animals for food in order to maintain their herds. However, more investigations are recommended to establish the intricate linkages between ECF and Iron deficiency anemia among women in pastoralist communities.

## 5 Conclusions

The findings from the study on anemia in children and mothers highlight critical risk factors that intertwine nutrition, education, and socioeconomic status. Notably, child age emerged as a significant determinant, with younger children exhibiting higher odds of anemia due to depleted iron stores and inadequate dietary sources. This is compounded by the nutritional status of both children and their mothers, where undernutrition correlates strongly with increased anemia risk. The study also reveals that maternal education plays a pivotal role; children of illiterate mothers are at a heightened risk, underscoring the importance of education in improving health outcomes through better nutrition knowledge and access to diverse diets. Additionally, the living conditions in pastoralist communities exacerbate these issues, as cultural practices and economic constraints limit access to essential healthcare and nutritious food.

Moreover, the research indicates that larger household sizes contribute to a greater likelihood of anemia due to resource competition and financial strain on families. The implications of maternal anemia during pregnancy further complicate the landscape, as it adversely affects birth outcomes and subsequent childhood health. The interplay between maternal education, household income, and dietary diversity emerges as a crucial framework for addressing anemia in these communities. Effective interventions should focus on promoting nutritional awareness for women, improving access to healthcare services, and child feeding practices, especially with animal source food consumption to mitigate the multifaceted risks associated with anemia in both mothers and children. Addressing these interconnected factors is essential for fostering healthier communities and reducing the prevalence of anemia in vulnerable populations.

## Author Contributions

**H. K. Wakhungu:** conceptualization (equal), formal analysis (equal), investigation (equal), methodology (equal), writing – original draft (equal), writing – review and editing (equal). **G. Abong:** supervision (lead), writing – original draft (equal), writing – review and editing (equal). **C. Muthike:** supervision (equal), writing – original draft (equal), writing – review and editing (equal). **J. Muema:** conceptualization (supporting), project administration (lead), supervision (equal), writing – original draft (equal), writing – review and editing (equal). **N. Mutono:** visualization (equal), writing – original draft (equal), writing – review and editing (equal). **G. P. Omondi:** methodology (equal), software (equal), writing – original draft (equal), writing – review and editing (equal). **S. M. Thumbi:** writing – original draft (equal), writing – review and editing (equal). **Z. Bukania:** conceptualization (equal), investigation (equal), supervision (supporting), writing – original draft (equal), writing – review and editing (equal).

## Conflicts of Interest

The authors declare no conflicts of interest.

## Data Availability

The data that support the findings of this study are available on request from the corresponding author.
